# Hoosier Sport: a research protocol for a multilevel physical activity-based intervention in rural Indiana

**DOI:** 10.3389/fpubh.2023.1243560

**Published:** 2023-07-27

**Authors:** Sarah J. Greeven, Paola A. Fernández Solá, Vanessa M. (Martinez) Kercher, Cassandra J. Coble, Katherine J. Pope, Temitope O. Erinosho, Aidrik Grube, Justin M. Evanovich, Nicole E. Werner, Kyle A. Kercher

**Affiliations:** ^1^Department of Kinesiology, School of Public Health-Bloomington, Indiana University, Bloomington, IN, United States; ^2^Department of Epidemiology and Biostatistics, School of Public Health-Bloomington, Indiana University, Bloomington, IN, United States; ^3^Department of Health and Wellness Design, School of Public Health-Bloomington, Indiana University, Bloomington, IN, United States; ^4^Department of Applied Health Science, School of Public Health-Bloomington, Indiana University, Bloomington, IN, United States; ^5^School of Education, University of Connecticut, Storrs, CT, United States

**Keywords:** human-centered design, youth, cardiovascular disease, multilevel intervention, physical activity, lifestyle intervention

## Abstract

**Introduction:**

Currently, only 1 in 4 children in the U.S. engage in the recommended amount of physical activity (PA) and disparities in PA participation increase as income inequities increase. Moreover, leading health organizations have identified rural health as a critical area of need for programming, research, and policy. Thus, there is a critical need for the development and testing of evidence-based PA interventions that have the potential to be scalable to improve health disparities in children from under-resourced rural backgrounds. As such, the present study utilizes human-centered design, a technique that puts community stakeholders at the center of the intervention development process, to increase our specific understanding about how the PA-based needs of children from rural communities manifest themselves in context, at the level of detail needed to make intervention design decisions. The present study connects the first two stages of the NIH Stage Model for Behavioral Intervention Development with a promising conceptual foundation and potentially sustainable college student mentor implementation strategy.

**Methods:**

We will conduct a three-phase study utilizing human-centered community-based participatory research (CBPR) in three aims: (Aim 1) conduct a CBPR needs assessment with middle school students, parents, and teachers/administrators to identify perceptions, attributes, barriers, and facilitators of PA that are responsive to the community context and preferences; (Aim 2) co-design with children and adults to develop a prototype multi-level PA intervention protocol called Hoosier Sport; (Aim 3) assess Hoosier Sport’s trial- and intervention-related feasibility indicators. The conceptual foundation of this study is built on three complementary theoretical elements: (1) Basic Psychological Needs mini-theory within Self-Determination Theory; (2) the Biopsychosocial Model; and (3) the multilevel Research Framework from the National Institute on Minority Health and Health Disparities.

**Discussion:**

Our CBPR protocol takes a human-centered approach to integrating the first two stages of the NIH Stage Model with a potentially sustainable college student mentor implementation strategy. This multidisciplinary approach can be used by researchers pursuing multilevel PA-based intervention development for children.

## Introduction

Cardiovascular disease (CVD) is the leading cause of death in the United States (US) and disproportionately impacts people from rural areas and lower socioeconomic backgrounds (1–4). While the impact of CVD is a critical public health issue, many of the risk factors for developing CVD are modifiable. Participating in physical activity (PA) is one of the most promising modifiable strategies to reduce CVD risk (5–7). However, currently only 1 in 4 children in the US engage in the recommended amount of PA (8). Furthermore, disparities in PA participation increase as income inequities increase (9). Since the progression of atherosclerosis begins in childhood and inactive children are likely to become inactive adults (10, 11), the promotion of PA should begin in childhood when prevention efforts may have optimal public health impact (12).

While the health consequences of physical inactivity affect all children, those from rural areas are disproportionately affected compared to urban children (13, 14). Children living within rural communities and from families with low-socioeconomic status (SES) often have less access and greater barriers to PA opportunities, lower health literacy, and less educational attainment, all of which are associated with lower PA participation rates and greater lifetime risk of developing CVD (4, 15, 16). Moreover, rural schools facing socioeconomic disadvantage are the least likely to offer policies (e.g., mandatory recess, economic development initiatives) and services (e.g., transportation, access to quality health services, and college and career readiness programs) that support PA programs (14, 17). Further, children from lower socioeconomic backgrounds are significantly more likely to feel unwelcome on school teams and not be able to afford to participate (16). Collectively, these challenges facing rural communities point to a critical need for intervention and align with the American Heart Association’s Presidential Advisory calling for rural populations to become a national priority for programming, research, and policy (15).

As rural populations continue to bear disproportionate burdens of disease and adverse health conditions, there has been growing support for developing and piloting of novel community-derived multilevel interventions (18–21). Multilevel interventions take a broader approach to intervening on complex health behaviors by targeting change at multiple levels of influence (e.g., individual, interpersonal, community) and have the potential to impact health outcomes more than single-level interventions (19–21). Accordingly, as suggested by the NIH Stage Model for Behavioral Intervention Development, behavioral interventions in PA need to be based on a strong empirical foundation (22). While knowledge of population level data is helpful, there needs to be greater emphasis on increasing specific understanding about how the PA-based needs of children from rural communities manifest themselves in context (NIH Stage 0), at the level of detail needed to make intervention design decisions (NIH Stage 1A) and in piloting interventions to determine feasibility (NIH Stage 1B). Taking a novel approach, the proposed study protocol places children at the center of each of the three study phases to get at the specific understanding necessary for targeted intervention development. A recent review of child-focused health research found that less than 1% of published studies included any form of advice from children during the research process (23). This general lack of inclusion of children in the research process occurs despite the recognized unique perspectives and ideas children can contribute that are otherwise unavailable to adult researchers (24, 25). Taken together, these research gaps point to the need for inclusion of children in promising multilevel PA intervention development and testing.

The powerful influence that college students can have on role modeling and supporting the behaviors of children is well recognized (26–28). For instance, children tend to view young adults as being more credible and relatable than older adults, having a better understanding of the concerns of young people, and being able to convey PA messages through interpersonal relationships (i.e., role modeling) to increase the likelihood of behavior change (29–31). Additionally, incorporating trained college student mentors as facilitators of intervention/programs may support cost-effectiveness and sustainability through reduced staffing costs and a consistent pipeline of incoming students. In community-based participatory research (CBPR), community stakeholders often express frustration with programs, particularly because the programs or interventions are short-term, provide little long-term benefit, and do not provide the needed infrastructure to sustain efforts (32). To address these frustrations, our implementation strategy using college student mentors takes a long-term approach to work with the community and builds capacity through ongoing college student mentor development and delivery of interventions/programming.

Therefore, the present study utilizes human-centered design, a technique that puts community stakeholders’ at the center of the intervention development and testing process (33). Human-centered design research is “*a systematic approach that holds empathy at its core and encourages its practitioners to return repeatedly to the context, emotions, needs, and desires of the key stakeholders they are developing their solutions for*” (34). Using a human-centered approach, the purpose of this three-phase study is to conduct a human-centered CBPR needs assessment (Aim 1; Stage 0), use participatory co-design with children and adults to develop a testable PA intervention protocol (Aim 2; Stage 1A), and to pilot/feasibility test the PA-based intervention, called *Hoosier Sport*, in a rural middle school (Aim 3; Stage 1B). Our primary hypotheses are that *Hoosier Sport* will be feasible as defined by multiple trial- and intervention-related feasibility indicators (e.g., recruitment capability, retention, fidelity, acceptability, attendance, compliance, cost, and appropriateness) (35). This formative work will be conducted to guide refinement and future testing of *Hoosier Sport* in a clinical trial.

## Methods and analysis

The following subsections describe the conceptual framework and each of the three phases of this study. Aim 1 is to conduct a community-based participatory research (CBPR) needs assessment with middle school students, parents, and teachers/administrators to identify perceptions, attributes, barriers, and facilitators of PA that are responsive to the community context and preferences. Aim 2 is to co-design with children and adults to develop a prototype multi-level PA intervention protocol called *Hoosier Sport*. Aim 3 is to assess *Hoosier Sport’s* trial- and intervention-related feasibility indicators in a sample of 6th grade middle school students. The present study defines PA in line with the Centers for Disease Control and Prevention as any bodily movement that is produced by the contraction of skeletal muscle and that substantially increases energy expenditure (36). We selected 6th grade students as the target population to balance the desire to intervene early in life (10, 11) with selecting a group that was mature enough for more advanced intervention strategies than elementary school students and aligned well with our research team’s behavioral expertise. Our overall hypothesis is that the three phases of the present study will lead to a feasible intervention protocol.

### Conceptual framework

**Figure 1:**
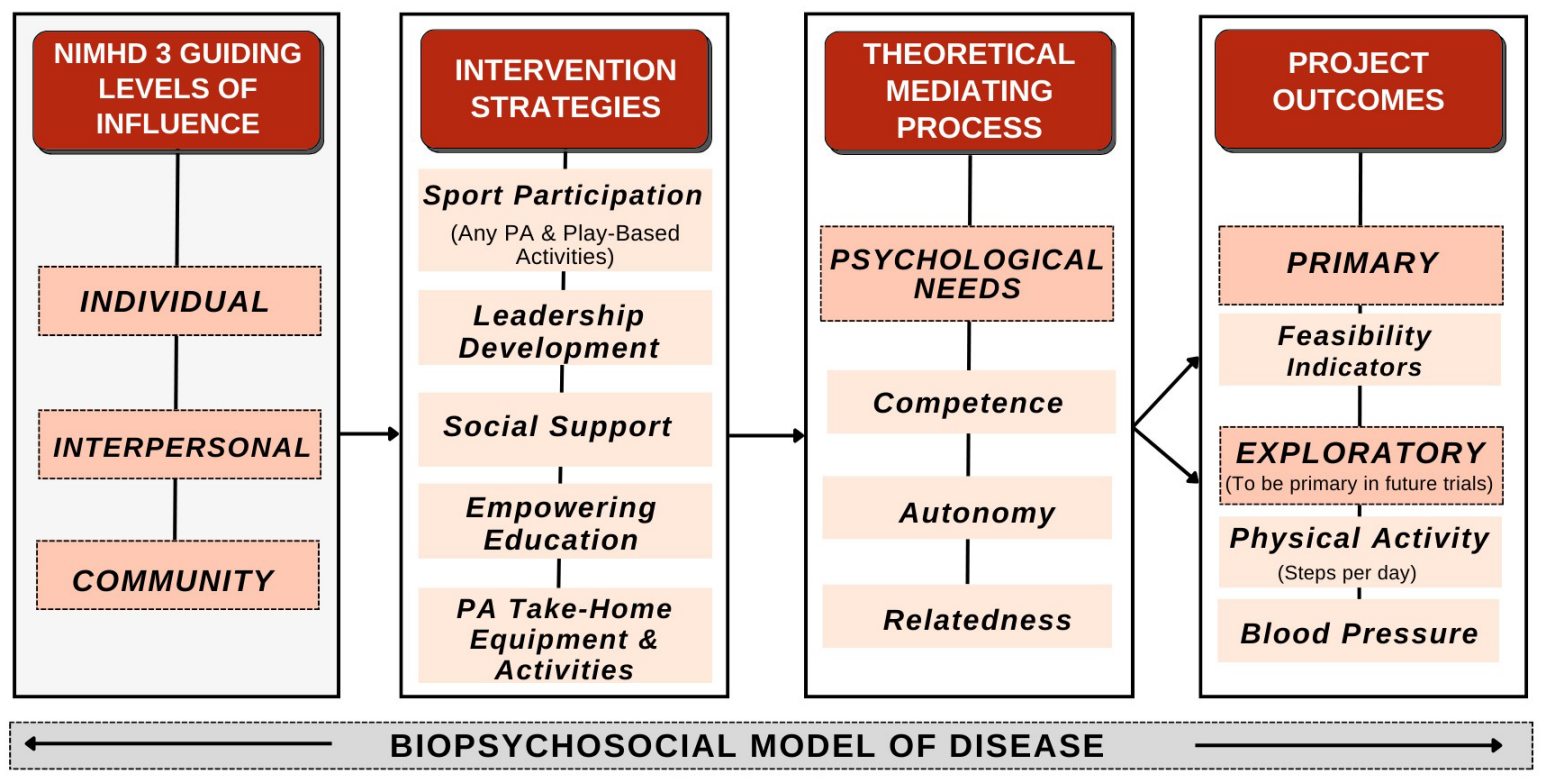
Conceptual framework.

The conceptual foundation of this study is built on three complementary theoretical elements (i.e., theory, model, and framework): (1) Basic Psychological Needs mini-theory within Self-Determination Theory (SDT) (37); (2) the Biopsychosocial Model (38); and (3) the National Institute on Minority Health and Health Disparities (NIMHD) Research Framework (39). These three conceptual models were each used to guide the methodology development for each of the three aims. Basic psychological needs will help the research team predict and examine the factors that influence our outcomes; the Biopsychosocial Model will support the description and interpretation of our findings but not predict outcomes; and the NIMHD Research Framework will help us conceptualize multilevel factors involved in understanding and reducing health disparities in our low-socioeconomic rural setting (Figure 1).

The first theoretical element of the present study, basic psychological needs, posits that increasing autonomy, competence, and relatedness will increase child well-being (37, 40–42). Inclusion of the psychological needs mini-theory will help guide our prediction of exploratory pilot/feasibility study outcomes (37). Aim 1 will include the validated Basic Psychological Needs in Exercise Scale to assess psychological needs within the exercise/PA context (43–45). Aim 2 co-design sessions include open-ended discussion questions designed to probe autonomy, competence, and relatedness. For example, an open-ended question targeting autonomy for children and adults will be “what physical activities are most appealing to you (your child)?”

The second theoretical element of the present study, the Biopsychosocial Model, allows for recognition of the interconnectedness between biological, psychological, and social factors in shaping an individual’s health and well-being (38). The Biopsychosocial Model informed our protocol development for our Aim 1 survey design, Aim 2 co-design guiding questions/themes, and the Aim 3 intervention evaluation plan. The Biopsychosocial Model will also help frame results within the larger biological, psychological, and social context. Specific examples of integrating the Biopsychosocial Model into the present study include designing and evaluating: (1) exploratory biological/physical outcomes (e.g., physical activity levels, blood pressure), (2) psychological components (e.g., psychometric scale evaluating autonomy, competence, and relatedness in PA context), and (3) social support strategies for PA (e.g., peer student and/or family support).

Lastly, the study also aligns with the NIMHD Research Framework that provides a system for targeting multiple levels of influence (e.g., individual, interpersonal, and community) (39). The PA-related barriers facing rural communities are complex and exist at multiple levels of influence. Utilizing the NIMHD Research Framework will assist Aim 3 assessment of progress, gaps, and opportunities. The following sections describe each Aim.

Aim 1 (Needs Assessment): Conduct a community-based participatory research (CBPR) needs assessment with middle school students, parents, and teachers/administrators to identify perceptions, attributes, barriers, and facilitators of PA that are responsive to the community context and preferences.

### Design

The study’s first phase is a CBPR needs assessment (NIH Stage 0) to identify the community’s physical activity-related needs, goals, opportunities, and assets. Despite knowledge of PA-based needs from a population level, we need to develop specific understanding of how the needs manifest themselves in context at the level of detail needed to make intervention design decisions. To conduct the PA-related needs assessment, we will survey children, parents, and teachers/administrators using a multi-level survey design targeting individual, interpersonal, and community levels of influence. This CBPR needs assessment will serve as a starting point for examining the PA context (e.g., school, home, weekdays, weekends) in the current school partner and inform future CBPR needs assessments with additional school partners.

### Setting and sample

We partnered with a rural Midwestern middle school with a population that is predominantly White and from low-SES backgrounds (the entire student body is eligible for free-and-reduced meals due to the school district’s high poverty). Data collection will include a survey sample of *n* = 40 students, *n* = 40 parents, and *n* = 15 teachers/administrators (total *n* = 95). The proposed sample size was selected to be feasible while having a large enough sample to have approximately normal distributions in our outcomes based on the central limit theorem (46–48). Inclusion criteria for children: (1) enrolled in the middle school; (2) entering 6th or 7th grade in fall 2023 semester; (3) have a parent/guardian willing to provide consent to participate; (4) willing to participate in the survey (assent). Inclusion criteria for parents/guardians: (1) Parent/guardian of a student currently enrolled at the school in 6th or 7th grade; (2) willing to participate in the survey. Inclusion criteria for teachers/administrators: (1) currently employed by the school; (2) willing to participate in the survey. We will purposively sample to ensure a balanced representation of male and female participants and diverse physical activity interests, including those who do not participate in much PA.

### Procedure

#### Child survey

After receiving consent from parents, we will obtain assent from children before they participate in the study to ensure children fully understand the assent document information, including the purpose of the study, study requirements, and potential risks or benefits. Parental consent will be collected remotely through an informed consent document distributed through Qualtrics survey software. Child assent and survey administration will be conducted through Qualtrics and occur in-person to increase compliance and understanding. The survey measures will include demographics, the Physical Activity Questionnaire for Children (PAQ-C) (49–52), Expanded Food and Nutrition Education Program (EFNEP) Food and Physical Activity Behaviors Questionnaire (53), Basic Psychological Needs in Exercise Scale (BPNES) (43, 44), and child-tailored/appropriate questions related to Policy-Systems-Environment (PSE) (54).

#### Parent/guardian survey (hereafter referred to as the adult survey)

For the adult survey, we will obtain consent and administer the survey remotely. Similar to the child survey, the adult surveys will include demographics, questions from the EFNEP Food and Physical Activity Behaviors Questionnaire (53), BPNES (43, 44), and select PSE questions from prior PA research (54). Both surveys will include pilot survey debriefing questions developed by survey methodologists from the Indiana University Center for Survey Research, encouraging participant feedback on survey methodology and assessing potential areas for improvement in future surveys. A more comprehensive understanding of the community’s PA landscape can be achieved by conducting separate surveys for the adults and children. See the *Measures* section for additional details and see Supplementary files 1, 2 for the complete child and adult surveys, respectively.

### Measures

#### Physical activity

The Physical Activity Questionnaire for Children (PAQ-C) will be used to assess self-reported physical activity behaviors in children (49, 52). The PAQ-C assesses PA during physical education class, recess, lunch, right after school, evening, weekends, and spare time. The PAQ-C consists of 10 items scored on a 5-point scale ranging from “*no*” activity being a 1 and “*7 times or more*” being a 5. In children, the PAQ-C has demonstrated good internal consistency, acceptable validity, and an adequate Cronbach’s alpha coefficient of 0.72–0.88 (50, 51). For the adult survey, PA-related questions are being asked about programming and PA equipment they would like to see offered at the school.

#### Nutrition

Questions from the Expanded Food and Nutrition Education Program (EFNEP) Food and Physical Activity Behaviors Questionnaire will be used to assess dietary intake. Questions covered nutritional behaviors “*over the last 7 days*” and “*yesterday*.” Of the original 30 questions on the questionnaire, the research team selected eight questions for children and 10 questions for adults to help ensure the survey will be feasible in terms of respondent burden. Response options allow participants to select how often they consume various food and drink options. The EFNEP began in 1969, serves all states and U.S. territories, and reaches 450,000 low-income youth and 200,000 low-income adults each year (53, 55). The EFNEP consistently shows more than 90% of adults and 80% of youth report improved nutritional practices (55, 56).

#### Psychological needs

Children and adults will rate the satisfaction of their psychological needs in exercise settings with the Basic Psychological Needs in Exercise Scale (BPNES). The BPNES measures psychological needs satisfaction in an exercise context based on autonomy, competence, and relatedness (43, 44, 57). The BPNES consists of 11 items with scores on a 5-point Likert scale ranging from “*I do not agree at all*” to “*I completely agree*.” Four items assessed autonomy, four for competence, and three for relatedness (57). In adults, the BPNES has demonstrated adequate internal consistency with Cronbach’s alpha coefficients of 0.84 for autonomy, 0.81 for competence, and 0.92 for relatedness, as well as acceptable discriminant and predictive validity (44). The scale scores are also largely unaffected by social desirability bias and have demonstrated stability over a 4-week period (44).

#### Policy-systems-environment

The adult survey will include questions addressing the PSE level of influence. Questions will assess adults’ interest in PA, nutrition, positive behavioral programming, and perceptions of current school PA policies and interest in new school PA policies. PA environmental questions were informed by past research on perceived environmental variables that may influence PA (54). As PA behaviors exist within an array of settings and levels of influence, questions focus on gaining an understanding of PA behaviors in various settings such as homes, neighborhoods, PA facilities, and parks. See Supplementary files 1, 2 for complete versions of the adult and child surveys, respectively.

### Data analysis

For descriptive statistics, we will compute frequencies and percentages for each categorical variable and calculate means and standard deviations for continuous variables. Quantitative analyses will be performed in R 4.0.3 (58). In collaboration with the Indiana University Center for Survey Research, the research team will review qualitative responses to identify general patterns and main themes. Results of the qualitative analysis will be reviewed by the research team and considered for incorporation into future school surveys. Results from Aim 1 will be used to inform Aim 2 co-design session topics and to guide open-ended question design.

Aim 2 (Participatory Co-design): Co-design with children and adults to develop a prototype multi-level PA intervention protocol, called *Hoosier Sport.*

### Design

**Figure 2:**
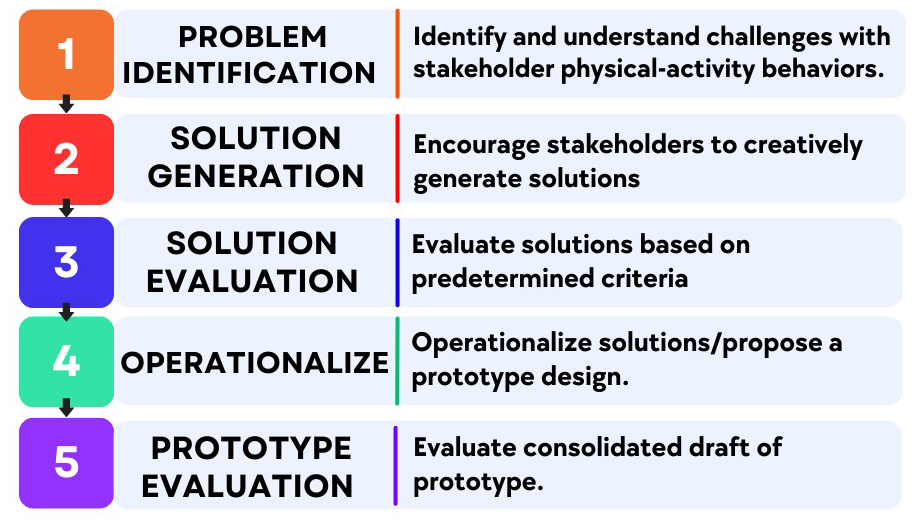
Participatory co-design sessions.

The study’s second phase is to co-design a prototype of the *Hoosier Sport* intervention protocol (NIH Stage 1A) to understand the unique PA-based needs of youth from primarily low-SES rural backgrounds by targeting individual, interpersonal, and school levels of influence. We will conduct a 5-step participatory co-design protocol that includes the following 5 session sequence: (1) problem identification; (2) solution generation; (3) solution evaluation; (4) operationalization; and (5) prototype evaluation. The participatory co-design process in our study context is designed to empower children and adults (i.e., parents/teachers/administrators) to provide input into the prototype *Hoosier Sport* PA intervention protocol. Based on preliminary school stakeholder input and previous PA intervention literature (59), the five preliminary intervention topics we anticipate designing are: (1) sport/PA participation (60); (2) leadership development (61); (3) social support for PA (62); (4) empowering education (63); (5) PA take-home equipment & activities (64). School administrators have requested that the *Hoosier Sport* intervention be designed to be conducted during physical education class. We will recruit two separate co-design teams, with one group consisting of *n* = 5 adults and one group of *n* = 5 children. Completion of the participatory co-design sessions will result in a testable prototype intervention protocol to be deployed in Aim 3. The intervention title *Hoosier Sport* was selected because *Hoosier* is a term of pride among many Indiana residents and integrating *sport* into the intervention is part of the “hook” to encourage children to participate in programming (Figure 2).

### Setting and sample

We will assemble two groups of co-designers, including one group of adults (parents, teachers/administrators) and one group of children, each with *n* = 5 individuals, which aligns with the standard range of participants needed for a participatory design (65). The odd number of participants allows for a majority vote to break ties between design alternatives within the group. Adults and children will be recruited via parent/guardian meetings and weekly newsletters distributed by school administrators. Due to the limited sample size and time commitment required for the co-design, we will use convenience sampling but will attempt to enroll approximately 50% female and 50% male and include participants who regularly participate in PA and those that do not. To be eligible for inclusion, children must be: (1) enrolled in the middle school; (2) entering 6th grade; (3) have parent/guardian consent to participate; (4) willing to participate in all 5 co-design sessions. To be eligible for inclusion, adults must be: (1) a parent/guardian of a student currently enrolled at the school in 6th or 7th grade or a teacher/administrator employed at the school; and (2) willing to participate in all 5 co-design sessions.

### Procedure

The two design teams will complete a series of five co-design sessions across 3 months, with approximately 2-weeks between sessions. The child group will begin the process, and in parallel, the adult group will have alternating sessions between the child sessions (e.g., child session, adult session, child session, etc.). The adult group will co-design with the study team to review and revise the child-developed prototype while striving to maintain as many child-derived components as possible. This parallel and alternating co-design process will allow children to have a sense of autonomy in the process to include important concepts to them (e.g., fun, enjoyment) while allowing the adults to refine the intervention protocol to increase the likelihood of feasibility and practicality.

The sessions will be facilitated by an experienced research team member with training in facilitating group coaching and discussions. The research team will develop open-ended questions to guide each session that are aligned with each session’s goals. For instance, in session 1, the design session agenda focuses on understanding challenges with children’s PA-related behaviors. The design process is an iterative process where we begin by coming to a common understanding of the challenges with PA-related behaviors, then collaboratively develop numerous divergent solution ideas for (1) sport/PA participation; (2) leadership development; (3) social support for PA; (4) empowering education; and (5) PA take-home equipment & activities. Then, we progressively move toward a detailed and high-fidelity intervention protocol. During each session, the facilitator will encourage discussion, interpretation, and respectful debate among design team members while ensuring progress. PA-based needs, goals, opportunities, and assets identified in Aim 1 will be integrated throughout the design session discussions by providing survey results to co-designers during session agenda development and finalization.

The research team will collect observation notes and audio recordings to analyze the design teams’ work as it is produced. These records will capture the co-design sessions, allowing for a detailed examination of the participants’ conversations, thought processes, and collaborative efforts in generating and grouping intervention design solutions. During the co-design sessions, the facilitators will help guide the participants’ conversation and thought process in generating and collaborating on intervention protocol design solutions. Each session will last for 60–90 min. Sessions will be audio recorded, and observation notes will analyze the design team’s work. In session 5, the teams will evaluate the prototype *Hoosier Sport* protocol feasibility, acceptability, and appropriateness on the Feasibility of Intervention Measure (FIM), Acceptability of Intervention Measure (AIM), and Intervention Appropriateness Measure (IAM) (66), each adapted to our study context and described in subsequent sections.

### Measures

The FIM, AIM, and IAM are four-item measures of implementation outcomes that are considered indicators of implementation success (66, 67). These measures can be used to prospectively determine the extent to which stakeholders believe *Hoosier Sport* will be feasible, acceptable, and appropriate (66, 67). The FIM, AIM, and IAM demonstrated adequate content validity, discriminant content validity, reliability, structural validity, structural invariance, and responsiveness to change (66).

### Data analysis

During the approximately 2-week periods between sessions, the research team will analyze the audio recording and observation notes of the sessions using the Rapid Identification of Themes from Audio-recordings (RITA) method (68). The RITA method allows for reliably coding and analyzing qualitative data without time-consuming transcription (68). We will apply descriptive statistics to analyze summative quantitative data from the FIM, AIM, and IAM used in session 5. At the conclusion of the analysis from session 5, we will have a detailed draft of an intervention protocol to pilot/feasibility test in Aim 3.

Aim 3 (Pilot Testing): Assess *Hoosier Sport’s* trial- and intervention-related feasibility indicators.

### Design

The third phase of the study is to assess intervention feasibility by testing the *Hoosier Sport* intervention with 6th grade students from one rural middle school twice per week for 8-weeks during physical education class (NIH Stage 1B). We will assess recommended trial- and intervention-related feasibility measures for pilot/feasibility studies (35). As exploratory outcomes, we will also assess PA levels (steps per day using Axivity AX3 accelerometers (69, 70)), blood pressure, and psychological needs using the BPNES (57). After the first pilot/feasibility test of *Hoosier Sport*, we will adopt a “traffic light” system of *a priori* progression criteria for feasibility outcomes (e.g., recruitment, retention) to guide intervention revisions and retesting *Hoosier Sport* the following semester. *Hoosier Sport* college student mentors will work alongside the research team to deliver the intervention (additional details in the next section). The college student mentors will be upper division undergraduate and graduate students enrolled in public health majors. To enhance intervention fidelity and as a part of the academic course, college student mentors will participate in 4-weeks of training classes prior to being deployed to the middle school, where they will receive course credit for hours spent delivering the intervention. Hypotheses for Aim 3 include the following: (3a) achieve full enrollment (*n* = 20); (3b) 85% retention at the end of the intervention; (3c) 75% attendance rate; (3d) mean score of ≥16 (a “good” score) on the FIM; (3e) mean score of ≥16 (a “good” score) on the AIM; (3f) mean scores of ≥16 (a “good” score) on the IAM; (3 g) 80% fidelity with intervention procedures.

### College student mentor implementation strategy for pilot/feasibility testing

*Hoosier Sport* college student mentors will be recruited through a service-learning course developed by the research team, titled “*Introduction to Youth Sport Development*,” and housed within the Department of Kinesiology at the Indiana University School of Public Health-Bloomington. This course includes topics such as effective mentoring techniques, communication strategies, and administering safety protocols. College student mentor models have been used to engage youth in PA, attain knowledge, and apply healthy behaviors, transferrable life skills, and academic enrichment (29–31). College students can provide personal support and guidance to overcoming environmental, social, and psychological barriers, leading to improved adherence to PA and increased peer resources to sustain PA (71, 72). Serving as role models, college students provide a dual intervention effect by gaining professional and practical experience while facilitating an environment that promotes positive youth development. Additionally, the constant influx of college students into the university has the potential to fulfill the delivery of PA-based interventions while being cost effective, potentially sustainable, and scalable (29–31).

### Setting and sample

We will recruit *n* = 20 6th grade students to participate in the pilot study. A sample size of at least 12 is considered adequate for intervention feasibility studies, but we will recruit more to account for potential dropout (48). The sample size of 20 was also selected within our resource limitations, initial staffing availability, and based on feasible recruitment estimates. Similar to Aim 2, we will use convenience sampling but strive for diversity of biological sex and physical activity participation. We will attempt to enroll approximately 50% female and 50% male participants and look to include participants who regularly participate in physical activity and those that do not. Inclusion criteria: (1) currently enrolled in 6th grade at the school; (2) have parental consent to participate; (3) agree to study participation (assent); (4) plan to attend all school days during the intervention period; (5) be available for baseline and post-intervention data collection.

### Procedure

The research team will introduce the initial study recruitment information via email and newsletter. A Qualtrics survey link with additional study information will be provided to those adults who express interest in having their child(ren) join the study. The research team will confirm eligibility via email/phone. Consent from parents to have their child(ren) join will populate a list of children who are eligible to be approached for assent. The research team will then host a study information and recruitment session for children at the school site. At this session, child participants will be provided with study information and a Qualtrics-based assent survey in appropriate and understandable terms for 6th grade students. Children will be provided with an opportunity to ask questions and informed that they can discontinue participation at any time during the study.

Enrolled participants will be mailed their initialized Axivity AX3 accelerometer (69, 70) and an instruction sheet. Participants’ PA will be collected using Axivity AX3 accelerometers for the 14 days at baseline (7 days pre-intervention and the first 7 days of the intervention) and another 14 days at the end of the intervention (the last 7 days of the intervention and 7 days immediately post-intervention). Participants who successfully return their accelerometer will be provided with their choice of sporting equipment (options: over-the-door basketball hoop, kick ball, or soccer ball). Blood pressure (BP) will be measured at the pre- and post-time points using an automatic Omron HEM 907XL blood pressure monitor (73) following American Heart Association protocols (e.g., three measurements 1 min apart, no caffeine or exercise within 30 min of assessment, and measuring at the same time each day). Participation will take place at their school during 2 days per week for 8-weeks. The pre- and post-data collection will take place during the lunch periods. The research team will schedule additional data collection for each time point to account for participants who miss the data collection event.

### Measures

In line with recently published NIH-funded pilot/feasibility research (35), we will assess two types of feasibility measures: (1) trial- and (2) intervention-related feasibility indicators. For trial-related feasibility indicators, we will measure recruitment capability and retention. For intervention-related feasibility indicators, we will measure treatment fidelity (i.e., assessing whether intervention components are delivered accurately, consistently, and with quality), acceptability, attendance, compliance (e.g., accelerometer usage), cost, and appropriateness (i.e., evaluating setting, cultural norms, or specific requirements). Measures will be collected at two time points (mid- and post-intervention) and recorded via Qualtrics using mobile devices.

#### Recruitment capability

Recruitment capability will be determined based on (1) the number of children successfully enrolled into *Hoosier Sport* (consent from parent/guardian and assent from child) and (2) the number of college students successfully enrolled into the “*Introduction to Sport-Based Youth Development*” course.

#### Retention

Retention will be measured based on (1) the number of children who participate in the post-intervention data collection event and (2) the number of college students who successfully completed their service-learning hours at the middle school. A make-up post-intervention event will be scheduled for child participants who miss the post-intervention data collection event.

#### Treatment fidelity

Three groups of stakeholders (children, college students, and the research team) will assess treatment fidelity using self-report measures at two time points (mid- and post-intervention) to explore whether the intervention was delivered accurately, consistently, and with quality. Assessment of fidelity will be guided by three questions from past school-based PA implementation research (74): (1) *to what extent was the intervention delivered as planned?* (2) *in what ways, if any, did the college student mentors adjust the program?* (3) *what were the reasons for any adjustments?*

#### Acceptability, appropriateness, feasibility

Children who participate in *Hoosier Sport* will rate the feasibility, acceptability, and appropriateness of the intervention using the FIM, AIM, and IAM, respectively (each described in Aim 2).

#### Compliance

Accelerometer compliance will be assessed for each of the two PA data collections by determining the number of days accelerometers collected data compared to the target.

#### Cost

Cost of the intervention will be monitored throughout the study period and determined in comparison to the prospective study budget.

Data analysis

#### Quantitative analysis of survey measures

For analysis, we will check the completeness and distributions of all variables. Normalizing transformations will be applied as needed for non-normally distributed variables. Internal consistencies of scaled scores are assessed with Cronbach’s alpha. Analyses of primary outcomes (i.e., feasibility indicators) and exploratory outcomes (i.e., PA, BP, BPNES) will be descriptive with means and standard deviations (SD).

## Discussion

Health interventions incorporating evidence and engaging key community members in the planning process generate more effective outcomes (34, 75, 76). As such, the World Health Organization has recognized human-centered design as a key strategy to address various health challenges and promote equitable healthcare solutions (77). Prior research has demonstrated that participatory co-design is an effective strategy for designing innovative interventions with unique populations (e.g., rural low-SES communities) (78, 79). Our human-centered protocol provides a foundation to yield a feasible intervention in accordance with Stages 0, 1A, and 1B of the NIH Stage Model for Behavioral Intervention Development. This protocol can be broadly applied by researchers who are developing and piloting PA-based interventions in schools.

The published participatory co-design approach employed by the present study has been shown to lead to effective intervention development (78, 80–82). Co-designed interventions are likely to be more engaging, satisfying, and useful to participants (83), and while co-design has been done in under-resourced PA contexts with children (84, 85), the field remains in its relative infancy. Our planned methods will consider the unique PA-related needs, goals, opportunities, and assets of rural children, parents, and teachers/administrators and are likely to lead to PA-based intervention that is uniquely responsive to the target middle school community. By including both trial- and intervention-related feasibility measures, the proposed protocol aligns with current literature on improving reporting of feasibility measures in pilot/feasibility studies (35) and may be more likely to lead to effectively informing future revisions to Stage 1B or subsequent Stage 2/3 efficacy clinical trials. A recent scoping review of behavioral pilot/feasibility studies found that *trial-related* feasibility was reported in many studies (i.e., recruitment and/or retention); however, important *intervention-related* feasibility indicators were not widely reported (i.e., fidelity, acceptability, attendance, compliance, cost, appropriateness) (35). Additionally, the implementation strategy utilizing a pipeline of college student mentors presents a potentially promising approach to addressing an often expressed weakness in CBPR research – frustration with programs, particularly because the programs/interventions are short-term, provide little long-term benefit, and do not provide the needed infrastructure to sustain efforts (32).

In sum, successfully completing the aims will lead to a feasible *Hoosier Sport* intervention poised for refinement or expansion in a Stage 2/3 efficacy clinical trial, powered to test changes in physical activity, with secondary outcomes of other cardiovascular disease risk factors (e.g., blood pressure, high-sensitivity C-reactive protein). Findings will also help inform other academic institutions practicing CBPR and aiming to partner with local schools. Ultimately, *Hoosier Sport* should be feasible and adaptable to a range of school contexts that could benefit immediately from partnerships with major academic institutions with the college student service-learning workforce to deliver programming at scale.

## Ethics statement

The studies involving human participants were reviewed and approved by Indiana University Institutional Review Board. Written informed consent for participation was not required for this study in accordance with the national legislation and the institutional requirements.

## Author contributions

SG and KK designed the initial study protocol and drafted the manuscript. SG, PF, VM, CC, KP, TE, AG, JE, NW, and KK contributed to the conceptualization and design of the study protocol. All authors contributed to the article and approved the submitted version.

## Funding

This work was supported by the SNAP-Ed grant program within the Division of Nutrition and Physical Activity at the Indiana Department of Health, as well as the Indiana University Office of the Vice Provost of Research, and the Indiana University Center for Innovative Teaching and Learning.

## Conflict of interest

The authors declare that the research was conducted in the absence of any commercial or financial relationships that could be construed as a potential conflict of interest.

## Publisher’s note

All claims expressed in this article are solely those of the authors and do not necessarily represent those of their affiliated organizations, or those of the publisher, the editors and the reviewers. Any product that may be evaluated in this article, or claim that may be made by its manufacturer, is not guaranteed or endorsed by the publisher.
