# Supplementary material for: Hoosier Sport: a research protocol for a multilevel physical activity-based intervention in rural Indiana
Source: Front Public Health. 2023 Jul 27;11:1243560. doi: 10.3389/fpubh.2023.1243560 (PMC10412824; doi:10.3389/fpubh.2023.1243560)
Supplement: Supplementary file 1 [file Data_Sheet_1.PDF]

Adult Survey

Yes, I agree to participate in this study.

- ○ No, I am **a parent/guardian** and do **NOT** wish to participate in this study.
- ○ No, I am **a teacher/administrator** (and **not** a parent) and do **NOT** wish to participate in this study.

____

Are you currently: *(Please select all that apply.)*

- ❑ A parent of a XX school student
- ❑ A teacher at XX school
- ❑ An administrator at XX school

The first several questions ask about the helpfulness of physical activity for a middle school child and what physical activities you might like to see offered at XX School.

How helpful do you believe physical activity (like playing sports, games, doing dance, or any other exercise) is for a middle school child's **physical health**?

- Not helpful at all
- A little helpful
- Moderately helpful
- Extremely helpful

How helpful do you believe physical activity (like playing sports, games, doing dance, or any other exercise) is for a middle school child's **social health** (for example, having good relationships with others)?

- Not helpful at all
- A little helpful
- Moderately helpful
- Extremely helpful

How helpful do you believe physical activity (like playing sports, games, doing dance, or any other exercise) is for a middle school child's **emotional health** (for example, being able to handle their emotions and feeling good about themselves)?

- Not helpful at all
- A little helpful
- Moderately helpful
- Extremely helpful

How do you feel about the amount of time that children get to be active at XX Middle School?

- Not even close to enough time
- Almost enough time
- Enough time
- A little bit too much time

- Way too much time

What sports or physical activities would you like to see offered for the first time or more often at XX Middle School? *Choose up to 3.*

- ❑ Badminton, tennis, or pickleball
- ❑ Walking or hiking for exercise
- ❑ Martial arts, like karate
- ❑ Soccer
- ❑ Bicycling
- ❑ Baseball or softball
- ❑ Volleyball
- ❑ Skateboarding
- ❑ Dance
- ❑ Football (include tag or tackle football)
- ❑ Tag
- ❑ Jogging or running
- ❑ Gymnastics
- ❑ Physical conditioning exercises, like jumping jacks, squats, or pushups
- ❑ Basketball
- ❑ Rowing, canoeing, or kayaking
- ❑ Ice hockey or ringette
- ❑ Swimming
- ❑ Other, please describe:_________________

If XX Middle School could have any new sports or physical activity equipment, what would you like the school to have? *Choose up to 3.*

- ❑ Clothing needed for sports or physical activities (examples: shorts, tights, sports bras)
- ❑ Strength training equipment (examples: dumbbells, weights, resistance bands)
- ❑ Sports balls for basketball, soccer, volleyball, etc.
- ❑ Nets for pickleball or badminton
- ❑ Basketball hoops
- ❑ Mats
- ❑ Bats/sticks/racquets
- ❑ Other, please describe:___________________

If students could learn anything about food, nutrition, or how to eat healthy, what would you like them to learn at XX Middle School? *Choose up to 3.*

- ❑ How to make healthy foods
- ❑ How to garden/grow healthy foods
- ❑ How to read labels on foods and what they mean
- ❑ How to look up things about food on their smartphone, tablet, or computer
- ❑ How to avoid food making them sick
- ❑ How much sugar is in different foods
- ❑ Where does the food we eat come from
- ❑ Other, please describe:_________________

If students could learn anything about how to do well in school or how to be a leader, what would you like them to learn at XX Middle School? *Choose up to 3.*

- ❑ How to start a business
- ❑ How to take care of mental health
- ❑ How to develop confidence
- ❑ How to find out what they are good at (their strengths)
- ❑ How to communicate better
- ❑ How to get more done (be productive)
- ❑ How to get into college or trade school
- ❑ How to listen to others
- ❑ How to study better
- ❑ How to get involved in after-school activities, sports, or clubs
- ❑ How to be organized
- ❑ How to have good relationships with other students
- ❑ How to be a leader or how to lead teams
- ❑ How to have good relationships with teachers
- ❑ Other, please describe:__________________

How interested are you in having **new physical activity or sports programs** implemented during the school day at XX Middle School?

- Not interested at all
- A little interested
- Moderately interested
- Extremely interested

How interested are you in having **new leadership programs** implemented during the school day at XX Middle School?

- Not interested at all
- A little interested
- Moderately interested
- Extremely interested

How interested are you in having **new nutrition programs** implemented during the school day at XX Middle School?

- Not interested at all
- A little interested
- Moderately interested
- Extremely interested

Do you think a new XX school policy should be created to help XX children be more physically active? (For example, recurring activity/walk breaks, more frequent physical education classes.)

- Yes
- No

What are the top barriers to physical activity participation for XX children? *Choose up to 3.*

- ❑ Lack of transportation
- ❑ Lack of interest
- ❑ Lack of support or encouragement from family, friends, or the community to participate in physical activity
- ❑ Student preference for doing other activities that don't involve physical activity, like being on their phone or watching TV
- ❑ Lack of sporting goods/ equipment
- ❑ Lack of facilities
- ❑ Lack of motivation/energy
- ❑ Cost of participating (for example, registration, travel fees)

The next couple of questions ask about areas where you live for middle school children to be involved in physical activity.

Which of the following do you think are problems where you live? *Choose as many as you wish.*

- ❑ There aren't enough places for children to be physically active near where we live.
- ❑ There aren't enough sports teams for children near where we live.
- ❑ The places to be physically active are run-down or don't have enough good equipment.
- ❑ I can't get my child to the places to be physically active or play sports easily.
- ❑ It costs too much for children to play sports.
- ❑ My child doesn't like organized sports teams.
- ❑ My child is not interested in sports or physical activity.

Now think about the trails, parks, recreational or fitness centers, or other places where children might walk, run, bike, or exercise near where you live. Which of the following do you think are problems where you live? *Choose as many as you wish.*

- ❑ There aren't enough places for children to walk, run, bike, or be physically active near where we live.
- ❑ There aren't enough physical activity groups for children where we live (for example, walking/running/biking groups).
- ❑ The places to walk, run, bike, or be physically active are run-down or don't have enough good equipment.
- ❑ I can't get my child to the places to walk, run, bike, or be physically active easily.
- ❑ It costs too much to have my child be part of a walking/running/biking group.
- ❑ My child is not interested in walking, running, biking, or being physically active.

The next few questions are about your level of physical activity during **your** free time.

During a **typical 7-day period (a week)**, how many times on average do you do **mild exercise requiring minimal effort, for example, easy walking, fishing from a river bank, bowling, or golfing without a cart**, for more than 15 minutes during your free time?

- 0 times per week
- 1-2 times per week
- 3-5 times per week
- 6-7 times per week
- 8-14 times per week
- 15 or more times per week

During a **typical 7-day period (a week)**, how many times on average do you do **moderate exercise that is not exhausting, for example, fast walking, easy bicycling, easy swimming, softball, or dancing**, for more than 15 minutes during your free time?

- 0 times per week
- 1-2 times per week
- 3-5 times per week
- 6-7 times per week
- 8-14 times per week
- 15 or more times per week

During a **typical 7-day period (a week)**, how many times on average do you do **strenuous exercise where your heart beats rapidly, for example, running, jogging, basketball, vigorous swimming, or vigorous long distance bicycling**, for more than 15 minutes during your free time?

- 0 times per week
- 1-2 times per week
- 3-5 times per week
- 6-7 times per week
- 8-14 times per week
- 15 or more times per week

The following questions ask how you feel about your exercise experiences in general. Please select the response that fits best with what you think or feel in general. *If a question does not apply, please select "Not applicable."*

Please select one response for EACH row.

	I don't agree at all	I agree a little bit	I somewhat agree	I agree a lot	I completely agree	Not applicable
I feel that I have made a lot of progress in relation to the exercise goal(s) I want to achieve.	○	○	○	○	○	○
The way I exercise is in agreement with my choices and interests.	○	○	○	○	○	○
I feel I perform successfully in the activity of my exercise program.	○	○	○	○	○	○
My relationships with the people I exercise with are friendly.	○	○	○	○	○	○
I feel that the way I exercise is the way I want to.	○	○	○	○	○	○
I feel exercise is an activity which I do very well.	○	○	○	○	○	○
I feel I have excellent communication with the people I exercise with.	○	○	○	○	○	○
I feel that the way I exercise is a true expression of who I am.	○	○	○	○	○	○
I am able to meet the requirements of my own exercise program.	○	○	○	○	○	○
My relationships with the people I exercise with are close.	○	○	○	○	○	○
I feel that I have the opportunity to make choices with regard to the way I exercise.	○	○	○	○	○	○

The next section asks you to think about what you had to eat and drink **yesterday**.

Yesterday, how many times did you eat vegetables, **not counting french fries**? *Include cooked vegetables, canned vegetables, and salads. If you ate 2 different vegetables in a meal or a snack, count them as 2 times.*

- None
- 1 time
- 2 times
- 3 times
- 4 or more times

Yesterday, how many times did you eat fruit? *Examples of fruits are apples, bananas, oranges, grapes, raisins, melon and berries. Includes fresh, frozen, dried, or canned fruit.* **Do not include juice.** *If you ate 2 different fruits in a meal or snack, count them as 2 times.*

- None
- 1 time
- 2 times
- 3 times
- 4 or more times

Yesterday, how many times did you drink sweetened drinks like soda, pop, fruit-flavored drinks, sports drinks, energy drinks, and vitamin water? *Do not include 100% fruit juice*.

- None
- 1 time
- 2 times
- 3 times
- 4 or more times

Now, for the next few questions, think about what you had to eat and drink during **the last 7 days.**

Over the last 7 days, how many days did you eat fruit? *Examples of fruits are apples, bananas, oranges, grapes, raisins, melon and berries. Include fresh, frozen, dried, or canned fruit.* ***Do not include juice.***

- I did not eat fruit
- 1 day
- 2 days
- 3 days
- 4 days
- 5 days
- 6 or 7 days

Over the last 7 days, how many days did you eat red or orange vegetables? *Examples of red or orange vegetables are tomatoes, red peppers, carrots, sweet potatoes, winter squash, and pumpkin.*

- I did not eat red or orange vegetables
- 1 day
- 2 days
- 3 days
- 4 days
- 5 days
- 6 or 7 days

Over the last 7 days, how many days did you eat dark green vegetables? *Examples of dark green vegetables are broccoli, spinach, dark greens, turnip greens, or mustard greens.*

- I did not eat dark green vegetables
- 1 day
- 2 days
- 3 days
- 4 days
- 5 days
- 6 or 7 days

Over the last 7 days, how many days did you drink sweetened drinks like soda, pop, fruit-flavored drinks, sports drinks, energy drinks, or vitamin water? *Do not include 100% fruit juice.*

- I did not drink any of these
- 1-3 days
- 4-6 days
- Every day

The next section has statements people have made about their food situation. Choose the answer that best fits your food situation over the last 30 days.

The food that I bought just didn’t last and I didn’t have money to get more.

- Often true
- Sometimes true
- Never true

I couldn't afford to eat balanced meals.

- Often true
- Sometimes true
- Never true

Did you ever cut the size of your meals or skip meals because there wasn't enough money for food?

- Yes
- No

Finally, we'd like to ask for some general background information about you.

Which category includes your age?

- 18-29
- 30-39
- 40-49
- 50-54
- 55+

What is your gender?

- Male
- Female
- I use a different term

Are you currently:

- Married or living with a partner
- Widowed
- Divorced

- Separated
- Never been married

What is your race or ethnicity? *Please select all that apply.*

- ❑ White
- ❑ Asian
- ❑ Middle Eastern or North African
- ❑ Black or African American
- ❑ Hispanic
- ❑ American Indian or Alaska Native
- ❑ Native Hawaiian or Other Pacific Islander

How many children 17 years of age or younger currently live in your household?

What is the highest degree or level of school you have completed?

- ○ Less than high school (no diploma, no GED or alternative credential)
- ○ High school graduate (with diploma, GED or alternative credential)
- ○ Some college credit, but no degree
- ○ Associate degree or trade school certificate (for example: AA, AS)
- ○ College graduate with a Bachelor's degree (for example: BA, BS)
- ○ Master's, doctoral, or professional degree (for example: MA, MS, EdD, PhD, JD, MD)

Please select the category below that includes the **total combined income, before taxes, for all members of your household for 2022.** Total household income includes money from jobs, net income from a business, farm, or a rental property, government assistance, and any other money income received by members of your household who are 15 years of age or older.

- ○ Less than $10,000
- ○ $10,000 - $19,999
- ○ $20,000 - $29,999
- ○ $30,000 - $44,999
- ○ $45,000 - $59,999
- ○ $60,000 - $74,999
- ○ $75,000 - $99,999
- ○ $100,000 or more
- ○ Prefer not to answer

During the past 12 months, was there a time that someone in your household needed to see a doctor (or other health care provider) but could not because you could not afford it?

- ○ Yes
- ○ No

Do you have any of the following? *Please select all that apply.*

- ❑ Deafness or difficulty hearing
- ❑ Blindness or difficulty seeing even when wearing glasses
- ❑ Not able to walk, climb stairs, or get up from a chair due to medical condition or age
- ❑ Difficulty concentrating, remembering, or making decisions
- ❑ Autism
- ❑ Learning disability
- ❑ Mental health condition, like anxiety or depression
- ❑ Medical condition, like asthma or diabetes

- [ ] None of the above

Please provide any additional comments or feedback you would like to share about your responses or this topic in general. *If none, please leave blank.*

Thank you for completing this survey! You are a part of a pilot. We would appreciate your feedback on a few additional questions to help us improve the study.

Please tell us about any questions that you didn't understand. *If none, please leave blank*

Please tell us about any questions that took you a long time or were hard to answer. *If none, please leave blank.*

Please tell us about any questions that were too personal or made you feel uncomfortable. *If none, please leave blank.*

How did you feel about how long the survey was?

- ○ Way too long
- ○ A little long
- ○ About right
- ○ A little short
- ○ Way too short

Please tell us about any technical problems you had completing the survey.
If none, please leave blank.
