# Supplementary material for: Hoosier Sport: a research protocol for a multilevel physical activity-based intervention in rural Indiana
Source: Front Public Health. 2023 Jul 27;11:1243560. doi: 10.3389/fpubh.2023.1243560 (PMC10412824; doi:10.3389/fpubh.2023.1243560)
Supplement: Supplementary file 2 [file Data_Sheet_2.PDF]

Child Survey

The first few questions ask about your free time when you're not in school.

During the school week, how many **hours a day** do you usually spend watching TV or movies, playing electronic or video games, or using a smartphone, tablet, or computer for something that is not schoolwork (for example, texting, using social media, or watching videos)?

- ○ None
- ○ 1 hour or less
- ○ 2 hours
- ○ 3 hours
- ○ 4 hours
- ○ 5 or more hours

During the weekend, how many **hours a day** do you usually spend watching TV or movies, playing electronic or video games, or using a smartphone, tablet, or computer for something that is not schoolwork (for example, texting, using social media, or watching videos)?

- ○ None
- ○ 1 hour or less
- ○ 2 hours
- ○ 3 hours
- ○ 4 hours
- ○ 5 or more hours

Which of the following activities do you like to do the most? *Choose up to 3.*

- ❑ Watching TV or movies
- ❑ Participating in the Scouts or 4H
- ❑ Doing volunteer or community service work
- ❑ Taking dance lessons
- ❑ Spending time on my smartphone, tablet, or computer
- ❑ Taking music or art lessons
- ❑ Going outside in the woods or to parks
- ❑ Playing video games
- ❑ Baking or cooking
- ❑ Participating in activities at my church
- ❑ Playing sports
- ❑ Doing after-school programs at school
- ❑ Spending time with my family

- ❑ Spending time with my friends
- ❑ Participating in school clubs
- ❑ Other, please describe:____________________

The next few questions are about what you think about physical activity and nutrition and what activities you might like to do **at school**.

How helpful do you believe physical activity (like playing sports, games, doing dance, or any other exercise) is for **good physical health, that is, how your body feels**?

- ○ Not helpful at all
- ○ A little helpful
- ○ Moderately helpful
- ○ Extremely helpful

How helpful do you believe physical activity (like playing sports, games, doing dance, or any other exercise) is for having **good relationships with your family, friends, teachers, and other people**?

- ○ Not helpful at all
- ○ A little helpful
- ○ Moderately helpful
- ○ Extremely helpful

How helpful do you believe physical activity (like playing sports, games, doing dance, or any other exercise) is for being **able to handle your emotions and feel good about yourself**?

- ○ Not helpful at all
- ○ A little helpful
- ○ Moderately helpful
- ○ Extremely helpful

How do you feel about the amount of time that you get to be physically active at your school?

- ○ Not even close to enough time
- ○ Almost enough time
- ○ Enough time
- ○ A little bit too much time
- ○ Way too much time

What sports or physical activities would you like to do more of at school? *Choose up to 3.*

Walking or hiking for exercise

- ❑ Swimming
- ❑ Basketball
- ❑ Bicycling
- ❑ Tag
- ❑ Martial arts, like karate
- ❑ Jogging or running
- ❑ Soccer
- ❑ Volleyball
- ❑ Skateboarding
- ❑ Badminton, tennis, or pickleball
- ❑ Baseball or softball
- ❑ Gymnastics
- ❑ Dance
- ❑ Ice hockey or ringette
- ❑ Physical conditioning exercises, like jumping jacks, squats, or pushups
- ❑ Rowing, canoeing, or kayaking
- ❑ Football (include tag or tackle football)
- ❑ Other, please describe:________________________

If you could have any new sports or physical activity equipment at school, what would you like to have? *Choose up to 3.*

- ❑ Nets for pickleball or badminton
- ❑ Basketball hoops
- ❑ Mats
- ❑ Clothing needed for sports or physical activities (example: shorts, tights, sports bras)
- ❑ Sports balls for basketball, soccer, volleyball, etc.
- ❑ Strength training equipment (examples: dumbbells, weights, resistance bands)
- ❑ Bats/sticks/racquets
- ❑ Other, please describe:_________________________

If you could learn anything about food, nutrition, or how to eat healthy, what would you like to learn? *Choose up to 3.*

- ❑ How much sugar is in different foods
- ❑ Where does the food we eat come from
- ❑ How to read labels on foods and what they mean
- ❑ How to garden/grow healthy foods
- ❑ How to make healthy foods
- ❑ How to avoid food making you sick
- ❑ How to look up things about food on my smartphone, tablet, or computer
- ❑ Other, please describe:________________________

If you could learn anything about how to do well in school or how to be a leader, what would you like to learn? *Choose up to 3.*

- ❑ How to get into college or trade school
- ❑ How to have good relationships with other students
- ❑ How to get involved in after-school activities, sports, or clubs
- ❑ How to be organized
- ❑ How to have good relationships with teachers
- ❑ How to find out what I'm good at (my strengths)
- ❑ How to listen to others
- ❑ How to develop confidence
- ❑ How to study better
- ❑ How to find a subject(s) in school that I like
- ❑ How to communicate better
- ❑ How to start a business
- ❑ How to be a leader or how to lead teams
- ❑ How to take care of my mental health
- ❑ How to get more done (be more productive) each day
- ❑ Other, please describe:________________________

How interested are you in having new **activities to help you learn how to be a good leader** during school hours?

- Not interested at all
- A little bit interested
- Moderately interested
- Extremely interested

How interested are you in having new **activities to help you learn about food and nutrition** during school hours?

- Not interested at all
- A little bit interested
- Moderately interested
- Extremely interested

How interested are you in having new **physical activity and sport-related activities** during school hours?

- Not interested at all
- A little bit interested
- Moderately interested
- Extremely interested

This next section is about your level of physical activity in **the last 7 days**. This includes sports or dance that make you sweat or make your legs feel tired, or games that make you breathe hard, like tag, jump rope, running, climbing, and others.

Please answer the questions honestly and to the best of your ability.

First, please think about what you did in your free time **in the last 7 days,** that is, when you were **not at school** and were spending time with your family, friends, or by yourself. **In the last 7 days,** how many times have you done each of the following activities for at least 10 minutes?

Please select one answer for EACH row.

Basketball	○	○	○	○	○
Ice hockey or ringette	○	○	○	○	○
Bicycling	○	○	○	○	○
Jogging or running	○	○	○	○	○
Martial arts, like karate	○	○	○	○	○
Gymnastics	○	○	○	○	○
Volleyball	○	○	○	○	○
Physical conditioning exercises, like jumping jacks, squats, or pushups	○	○	○	○	○
Football (include tag or tackle football)	○	○	○	○	○
Walking or hiking for exercise	○	○	○	○	○
Swimming	○	○	○	○	○
Baseball or softball	○	○	○	○	○
Skateboarding	○	○	○	○	○
Soccer	○	○	○	○	○
Dance	○	○	○	○	○
Rowing, canoeing, or kayaking	○	○	○	○	○
Tag	○	○	○	○	○
Badminton, tennis, or pickleball	○	○	○	○	○
Other, please specify: ______	○	○	○	○	○

In the last 7 days, on how many days **right after school,** did you do sports, dance, or play games in which you were very active? *Remember that "very active" means activities that make you sweat or make your legs feel tired, or games that make you breathe hard, like tag, skipping, running, climbing, and others.*

- None
- 1 time
- 2 or 3 times
- 4 times
- 5 times

In the last 7 days, on how many **evenings**, did you do sports, dance, or play games in which you were very active? *Remember that "very active" means activities that make you sweat or make your legs feel tired, or games that make you breathe hard, like tag, skipping, running, climbing, and others.*

- None
- 1 time
- 2 or 3 times
- 4 or 5 times
- 6 or 7 times

Last weekend, how many times did you do sports, dance, or play games in which you were very active?

- None
- 1 time
- 2-3 times
- 4-5 times
- 6 or more times

Which one of the following describes you best for **the last 7 days**? Read **all five** statements before deciding on the **one** answer that describes you.

- All or most of my free time was spent doing things that involve little physical effort.
- I did physical things in my free time 1-2 times in the last 7 days (e.g. play sports, went running, swimming, bike riding, did exercises).
- I did physical things in my free time 3-4 times in the last 7 days.
- I did physical things in my free time 5-6 times in the last 7 days.
- I did physical things in my free time 7 or more times in the last 7 day.

Now, think about your time spent **at school during the last 7 days**.

In the last 7 days, during your physical education (PE) classes, how often were you very active (playing hard, running, jumping, throwing)?

- Hardly ever
- Sometimes
- Quite often
- Always
- I don't do PE

In the last 7 days, what did you normally do **at lunch**?

- Sat down (talking, reading, doing schoolwork)
- Stood around or walked around
- Ran or played a little bit
- Ran around and played quite a bit
- Ran and played hard most of the time

In the last 7 days, what did you do most of the time during **any other free time you had at school (e.g., recess, reward period, etc.)?**

- Sat down (talking, reading, doing schoolwork)
- Stood around or walked around
- Ran or played a little bit
- Ran around and played quite a bit
- Ran and played hard most of the time
- I didn't have any other free time at school (e.g., recess, reward period, etc.)

Were you sick at any time during the last 7 days, or did anything prevent you from doing your normal physical activities?

- Yes
- No

The following questions ask about how you feel about your exercise experiences in general, including both at school and in your free time.

Please select one response for EACH row.

	I don't agree at all	I agree a little bit	I somewhat agree	I agree a lot	I completely agree
The way I am physically active is the way I want to be.	○	○	○	○	○
I do well in the activities I do for exercise.	○	○	○	○	○
The way I am physically active is just right for someone like me.	○	○	○	○	○
I can easily talk to the people I am physically active with.	○	○	○	○	○
Physical activity is something I do very well.	○	○	○	○	○
I feel close to the people I am physically active with.	○	○	○	○	○
I'm getting what I want to get out of physical activity in my life.	○	○	○	○	○
I have good relationships with the people I am physically active with.	○	○	○	○	○
The way I am physically active fits with my choices and interests.	○	○	○	○	○
I can do what I need to do to get what I want out of physical activity in my life.	○	○	○	○	○
I get to make choices about how I want to be physically active.	○	○	○	○	○

The next section asks you to think about what you had to eat and drink **yesterday**.

Yesterday, how many times did you eat vegetables, **not counting french fries**? Include cooked vegetables, canned vegetables, and salads. *If you ate 2 different vegetables in a meal or a snack, count them as 2 times.*

- None
- 1 time
- 2 times
- 3 times
- 4 or more times

Yesterday, how many times did you eat fruit? Examples of fruits are apples, bananas, oranges, grapes, raisins, melon and berries. Include fresh, frozen, dried, or canned fruit. **Do not include juice**. *If you ate 2 different fruits in a meal or snack, count them as 2 times.*

- None
- 1 time
- 2 times
- 3 times
- 4 or more times

Yesterday, how many times did you drink sweetened drinks like soda, pop, fruit-flavored drinks, sports drinks, energy drinks, or vitamin water? *Do not include 100% fruit juice.*

- None
- 1 time
- 2 times
- 3 times
- 4 or more times

Now, for the next few questions, think about what you had to eat and drink during **the last 7 days.**

Over the last 7 days, how many days did you eat fruit? *Examples of fruits are apples, bananas, oranges, grapes, raisins, melon and berries. Include fresh, frozen, dried, or canned fruit.* ***Do not include juice.***

- I did not eat fruit.
- 1 day
- 2 days
- 3 days
- 4 days
- 5 days
- 6 or 7 days

Over the last 7 days, how many days did you eat red or orange vegetables? *Examples of red or orange vegetables are tomatoes, red peppers, carrots, sweet potatoes, winter squash, and pumpkin.*

- I did not eat red or orange vegetables.
- 1 day
- 2 days
- 3 days
- 4 days
- 5 days
- 6 or 7 days

Over the last 7 days, how many days did you eat dark green vegetables? *Examples of dark green vegetables are broccoli, spinach, dark greens, turnip greens, or mustard greens.*

- I did not eat dark green vegetables.
- 1 day
- 2 days
- 3 days
- 4 days
- 5 days
- 6 or 7 days

Over the last 7 days, how many days did you drink sweetened drinks like soda, pop, fruit-flavored drinks, sports drinks, energy drinks, or vitamin water?

- I did not drink sweetened drinks like soda, pop, fruit-flavored drinks, sports drinks, energy drinks, or vitamin water.
- 1-3 days
- 4-6 days
- Every day

Over the last 7 days, how many times did you drink sweetened drinks like soda, pop, fruit-flavored drinks, sports drinks, energy drinks, or vitamin water **each day that you drank them**?

- 1 time a day
- 2 times a day
- 3 times a day
- 4 or more times a day

The last few questions ask for some general background information about you.
How old are you?

- 9 years old or younger
- 10 years old
- 11 years old
- 12 years old
- 13 years old or older

What grade will you be going into this coming fall?

- 5th grade
- 6th grade
- 7th grade

What is your gender?

- Female
- Male
- I use a different term

What is your race or ethnicity? *Please select all that apply.*

- ❑ White
- ❑ Asian
- ❑ Middle Eastern or North African
- ❑ Black or African American
- ❑ Hispanic
- ❑ American Indian or Alaskan Native
- ❑ Native Hawaiian or Other Pacific Islander

Do you have any of the following? *Choose all that you have.*

- ❑ Deafness or difficulty hearing
- ❑ Blindness or difficulty seeing even when wearing glasses
- ❑ Not able to walk, climb stairs, or get up from a chair due to a medical condition
- ❑ Difficulty concentrating, remembering, or making decisions
- ❑ Autism
- ❑ Learning disability
- ❑ Mental health condition, like anxiety or depression
- ❑ Medical condition, like asthma or diabetes
- ❑ I don't have any of these

Please tell us anything else you'd like to about your school or your life. If you don't have anything else to say, just leave it blank.

Thank you for completing this survey! You are a part of a pilot. We would appreciate your feedback on a few additional questions to help us improve the study. *If none, please leave blank.*

Please tell us about any questions that you didn't understand.

Please tell us about any questions that took you a long time or were hard to answer.

Please tell us about any questions that were too personal or made you feel uncomfortable.

How did you feel about how long the survey was?

- ○ Way too long
- ○ A little long
- ○ About right
- ○ A little short
- ○ Way too short

Please tell us about any technical problems you had completing the survey.
